# Trends in oncology marketing applications in the European Union: a five‐year systematic review

**DOI:** 10.3389/fphar.2025.1738701

**Published:** 2026-01-20

**Authors:** Ricardo Basto, Siddartha Karaya, Alex Zwiers

**Affiliations:** Zwiers Regulatory Consultancy, a Part of ProductLife Group, Oss, Netherlands

**Keywords:** Accelerated assessment, scientific advice, cancer, conditional marketing authorization, drugs, European Union, and Orphan designation

## Abstract

In the European Union, before cancer medicines containing a new active substance become available to patients, they must undergo a rigorous authorization process through the Centralized Procedure. This study examines trends in oncology Marketing Authorization Applications (MAAs) over the past 5 years. Oncology MAAs from January 2020 to January 2025 were used to collect and analyze publicly available data. The number of MAAs, therapeutic indications, company and product characteristics, scientific advice (SA), orphan designation (OD)/expedited programs, and overall procedural time were analyzed. A total of 60 MAAs were identified. During the reporting period, only three MAAs received negative opinions; however, two were reverted to positive after re-examination. Blood cancers were the main therapeutic indication. The typical profile of a MAA was as follows: a large-sized company holder (72%); monoclonal antibody (mAb) (31%); SA requested (92%); under OD at both time points: at the moment of application (60%) and at the moment of receiving the opinion by the Committee for Medicinal Products for Human Use (67% of the initial number of MAAs under OD); not granted any expedited program (57%); and with an average total procedure time of about 348 days. Additionally, procedural time analysis revealed shorter timelines for MAAs under the accelerated assessment (AA) program and those that obtained SA. Oncology MAAs have increased over the years, particularly for mAbs and blood cancer indications. Large-sized companies were the main MAA holders. Additionally, the SA and AA program might have demonstrated a positive impact in reducing procedural time.

## Graphical Abstract

**Figure:**
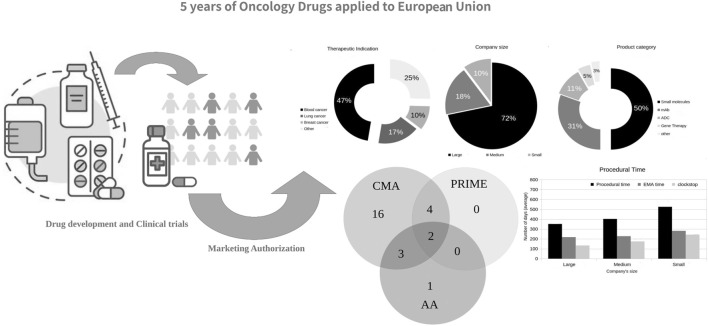

## 1 Introduction

Cancer is a disease characterized by the uncontrolled growth of transformed cells that evolve through natural selection (Brown et al., 2023). According to the World Health Organization (WHO), approximately 1 in 6 deaths is caused by cancer. It is the second leading cause of death globally, with its burden continuing to grow and placing substantial physical, emotional, and financial strain on individuals, families, communities, and healthcare systems (WHO, 2025). Up to date, companies seeking marketing approval for medicines focused to treatment of cancer in the European Union (EU) and in the European Economic Area (EEA) must submit a Marketing Authorization Application (MAA) to the European Medicines Agency (EMA) through the Centralized Procedure (CP). The CP is ruled by regulation European Commission (EC) 726/2004, which is coordinated by the EMA followed by authorization granted by the EC (European Commission, 2019).

In medicine development, patients with serious diseases and unmet medical needs often face long waits, as both investigative therapy development and health authority review processes can feel unreasonably slow (Vallano et al., 2023). In light of this, the EMA has established special status and expedited programs to encourage and/or to accelerate both drug development and the review of MAAs for promising therapies targeting serious diseases and unmet medical needs. These include orphan designation (OD), priority medicines (PRIME), conditional marketing authorizations (CMA) (or exceptional circumstances–EXC), and accelerated assessment (AA). OD supports the development of treatments for rare diseases through regulatory and financial incentives (Fermaglich and Miller, 2023). The PRIME program facilitates the development of treatments for unmet medical needs by allowing early and active interaction with regulatory authorities (Alaburde et al., 2025). CMA and EXC seek to accelerate patient access to treatments for unmet medical needs by moving certain stages of the drug development process to after approval; however, EXC is approached when comprehensive data are not available (Maksimova et al., 2024). MAAs under AA program have the procedural time reduced through a faster, but no less rigorous, evaluation (Del Grosso et al., 2025). In addition, the availability of a guidance, known as scientific advice (SA) or as protocol assistance (in case of MAA under OD), throughout the development and submission phases helps facilitate and streamline the process (EMA, 2025a).

Previous studies have analyzed medicines applications in the EU over specific periods (Regnstrom et al., 2010; Grössmann et al., 2021; Costa Gonçalves et al., 2022; Ruuskanen et al., 2024). However, there is a lack of recent, comprehensive, and systematic studies evaluating the characteristics of MAA procedures of new medicines for cancer therapy, particularly regarding the impact of SA, OD and expedited programs on the procedural time. Therefore, the present study aims to identify trends in oncology MAAs over the past 5 years, highlighting procedural time, SA, OD and expedited programs, taking into account company sizes and product categories.

## 2 Methodology

The present method was performed according to Garsen et al. (2021), with some modifications. In addition, Preferred Reporting Items for Systematic Reviews and Meta-Analysis (PRISMA) guidelines have been followed (Moher et al., 2009).

### 2.1 Data collection and eligibility criteria

**FIGURE 1:**
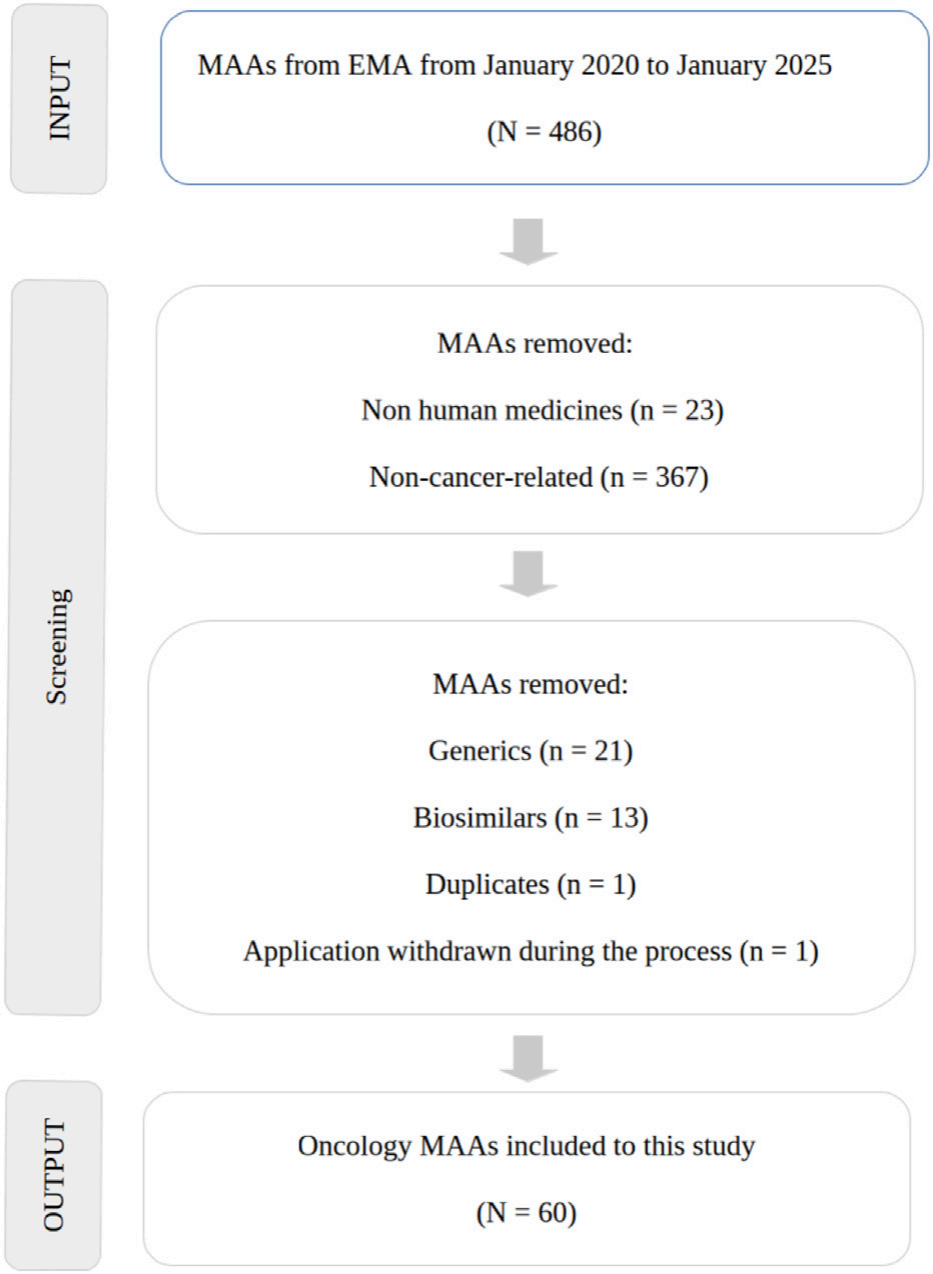
PRISMA flow diagram of CP identified and included for the review of EPARs.

Briefly, the output from January 2020 until January 2025 of cancer therapy applications with either positive or negative Committee for Medicinal Products for Human Use (CHMP) opinions were automatically generated from the human medicine highlights published by EMA^1^. In addition, European Public Assessment Reports (EPARs) were used as the primary source of information. As inclusion criteria, only oncology MAAs with product classified as a new active substance and article 8 (3) full or full mixed applications under the Directive 2001/83/EC as legal basis were used in this study (European Parliament and Council of the European Union, 2001). As the exclusion criteria, MAA withdrawn by the applicant before receiving opinion, and products related to diagnostic or non-direct action against cancer (only acting on the reduction of side or secondary effects) were removed from this study (Figure 1).

### 2.2 Data analysis and presentation

Initially, the number of MAAs with a CHMP opinion were counted and separated according to the year and their respective therapeutic indication, and their respective legal MAA holder. Based on the number of employees, companies were defined and categorized differently from EMA SME definition as small (not more than 500), medium (between 500 and 5,000), or large-sized companies (more than 5,000) (Garsen et al., 2021). Additionally, the holder’s status in the EMA SME Register was assessed (EMA, 2025b). The MAAs were classified in relation to product per category according to their characteristics as biotechnology-related products (monoclonal antibody - mAb, antibody drug-conjugate–ADC, gene therapy, and others) or small molecules. The MAAs that benefited from SA were identified and analyzed, along with the number of meetings held and the aspects discussed considering holder’s and product’s features, respectively. MAAs under OD and/or expedited program(s) (i.e., AA, CMA/EXC, and PRIME) were verified and counted considering holder’s and product’s features. To determine the impact of SA and AA program on the procedural time, the period that the applicant had to submit their responses to the first list of questions (LoQ, as known as first clockstop), as well as the procedural time (total procedure time, EMA time, and clockstop time) were analyzed in MAAs granted and not granted SA and AA, considering holder’s and product’s features. The results were shown as categorical variables, which were described with absolute (n) and relative frequency (%). In addition, MAA holders and product names were not disclosed to avoid conflict of interests.

## 3 Results

### 3.1 Trends and key features of oncology MAAs

**FIGURE 2:**
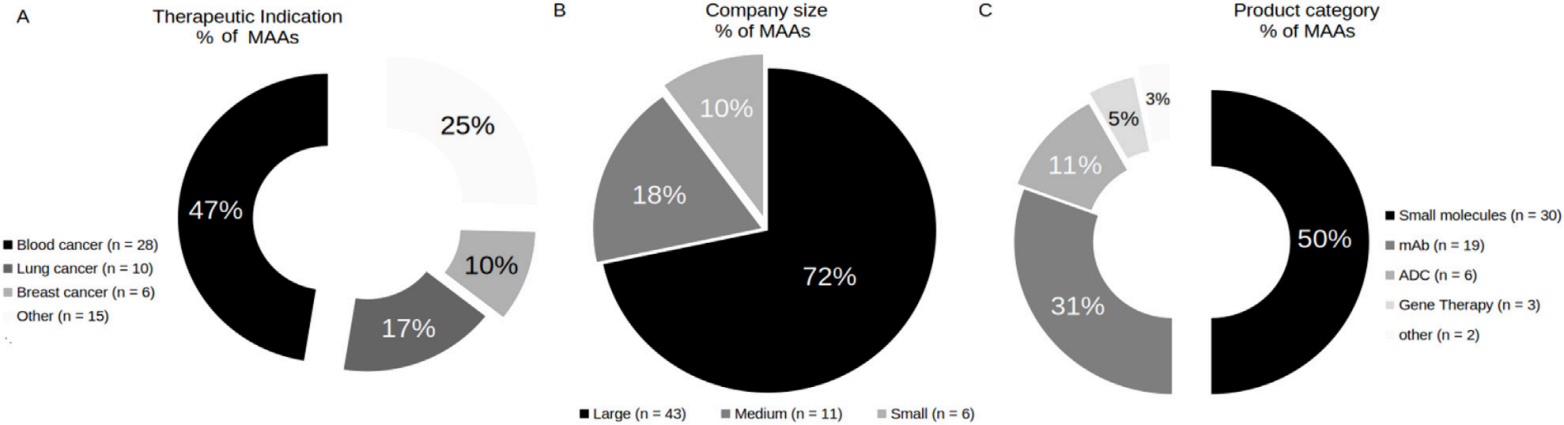
Classification of MAAs related to therapeutic indication **(A)**, company size **(B)** and product category **(C)**.

A total of 60 oncology MAAs with CHMP opinion, or an average of 12 MAA per year, were found between January 2020 and January 2025. From those, three (5%) of MAAs obtained a negative CHMP opinion. However, two (3%) obtained a positive CHMP opinion after re-examination of the procedure. Considering therapeutic indications, only products with a positive opinion from the CHMP (n = 59) were analyzed. Among them, blood cancer (47%, n = 28), lung cancer (17%, n = 10), and breast cancer (10%, n = 6) accounted for the majority. Other cancers included gastrointestinal cancer (7%, n = 4), ovarian and peritoneal cancer (5%, n = 3), skin cancer (5%, n = 3), bladder cancer (3%, n = 2), prostate cancer (2%, n = 1), hepatic cancer (2%, n = 1), and gallbladder cancer (2%, n = 1) (Figure 2A). Among of MAAs, 34 application holders were identified. Considering the number of MAAs per holder, only 8 MAA holders accounted for 50% of the total of oncology MAAs over the period analyzed, with one of them being a medium-sized companies (data not shown). Of the 60 MAAs, 72% (n = 43) were submitted by large-sized company holders, 18% (n = 11) by medium-sized company holders, and 10% (n = 6) by small-sized company holders (Figure 2B). Between medium and small-sized company holders, only one small-sized company holders (4%, n = 1) was found registered in EMA SME register. Regarding the product categories involved, there was an equilibrium (50%/50%) in MAAs with biotechnology-related products and small molecules involved (Figure 2C). Among the biotechnology-related products, the majority were classified as mAbs (31%, n = 19), followed by ADCs (11%, n = 6), and gene therapy products (5%, n = 3). Additionally, one MAA with fusion protein with high molecular weight as a product, and one MAA with a bi-specific antibody were identified and both classified as “other” (3%, n = 2).

### 3.2 Overview of SA distribution by holder’s and product’s features

**FIGURE 3:**
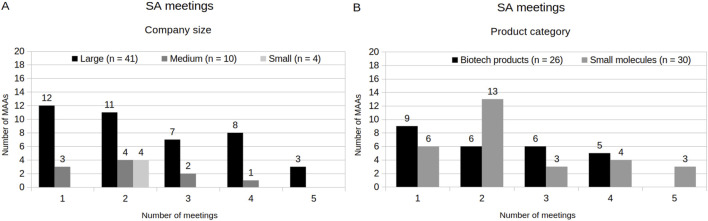
Scientific Advice. **(A)** SA meetings by company size. **(B)** SA meetings by Product category.

92% of MAAs (n = 55) obtained SA. Among these, 75% (n = 41) of the MAAs had large-sized company holders, while 18% (n = 10) and 7% (n = 4) had medium- and small-sized company holders, respectively. Regarding product category, MAAs with products with small molecules involved obtained slightly more SA (53%, n = 29) than those with biotechnology-derived products involved (47%, n = 26). In addition, the number of SA meetings per MAA ranged from one to five, depending on company size holders and product type involved (Figures 3A,B). MAAs with small-sized company holders held two meetings with SA group, while MAAs with large- and medium-sized company holders showed greater variability (from 1 to 5 meetings). Additionally, MAAs with small molecules products involved exhibited the most variation, with the majority holding two meetings per application.

### 3.3 Distribution and profile of MAAs under OD and expedited programs

#### 3.3.1 Orphan designation (OD)

**FIGURE 4:**
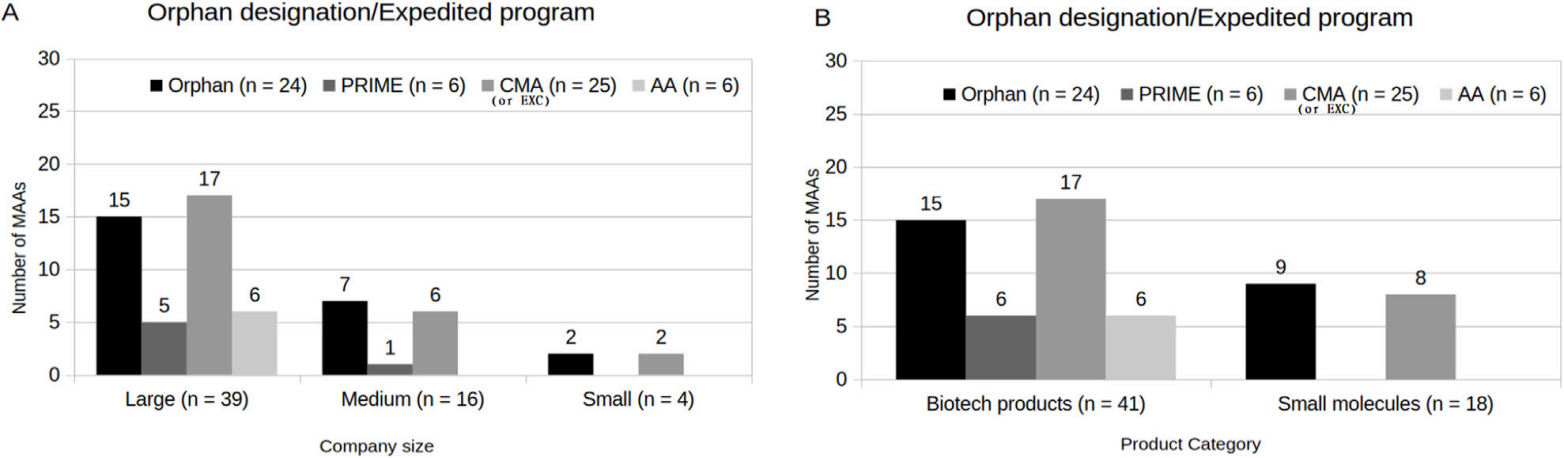
Orphan designation/Expedited program(s) (AA, CMA/EXC and/or PRIME). **(A)** Company size. **(B)** Product category.

Concerning the MAAs indicated for rare cancers, 60% (n = 36) obtained OD from the beginning of the application. The indications consisted mainly of blood cancers (75%, n = 27). However, only 67% of those (n = 24) maintained this designation until the end, followed by a CHMP opinion. Of these remaining MAAs under OD, most of them were indicated for blood cancers (59%, n = 16). As a minority, there were MAAs under OD also indicated for skin (7%, n = 2), gastrointestinal (4%, n = 2), ovarian and peritoneal (4%, n = 1), esophageal (4%, n = 1), and lung (4%, n = 1) cancers, respectively. In addition, one product (4%, n = 1) received a negative opinion for the following indication: tenosynovial giant cell tumour (TGCT). Regarding MAA’s holders, the majority were represented by large-sized companies (63%, n = 15) (Figure 4A). Medium and small-sized company holders represented 29% (n = 7) and 8% of the MAAs (n = 2) under OD, respectively. Considering the relation between OD and product characteristics, 63% of the MAAs (n = 15) had biotechnology-related products and 37% (n = 9) had small molecules involved (Figure 4B).

#### 3.3.2 Priority medicines (PRIME)

In relation to MAAs with PRIME status, only 10% (n = 6) were granted under the PRIME program. 83% of the MAAs (n = 5) had large-sized company holders, and only 17% of the MAAs (n = 1) had medium-sized company holders. Furthermore, all of them were biotechnology-related products. No MAA with a small-sized company holder was granted with PRIME.

#### 3.3.3 Accelerated assessment (AA)

With respect to the MAAs under AA program, 37% (n = 22) had initially requested this pathway; however, only 64% of these (n = 14) were granted. Among them, 57% (n = 8) were reverted to the standard procedure during the evaluation process. The reasons were related to the need for additional clarification or data, issues identified by the committee that require further analyzes, need of additional studies, or detailed clarifications that could not be provided within the accelerated timeline. Ultimately, only 43% of the MAAs initially granted AA (n = 6) obtained a CHMP opinion under the AA. Additionally, all MAAs that concluded the process under AA were from large-sized companies and involved biotechnology-related products.

#### 3.3.4 Conditional marketing authorizations (CMA) and exceptional circumstances (EXC)

38% of the MAAs (n = 23) were granted CMA, and 3% (n = 2) were authorized under EXC. Regarding MAA holder’s size, 68% (n = 17) were large-sized companies, 24% (n = 6) medium-sized companies, and 8% (n = 2) small-sized companies. By product type, 68% (n = 17) of the MAAs granted CMA or EXC were biotechnology-derived products, while 32% (n = 8) were small molecules.

#### 3.3.5 Independent variables

**FIGURE 5:**
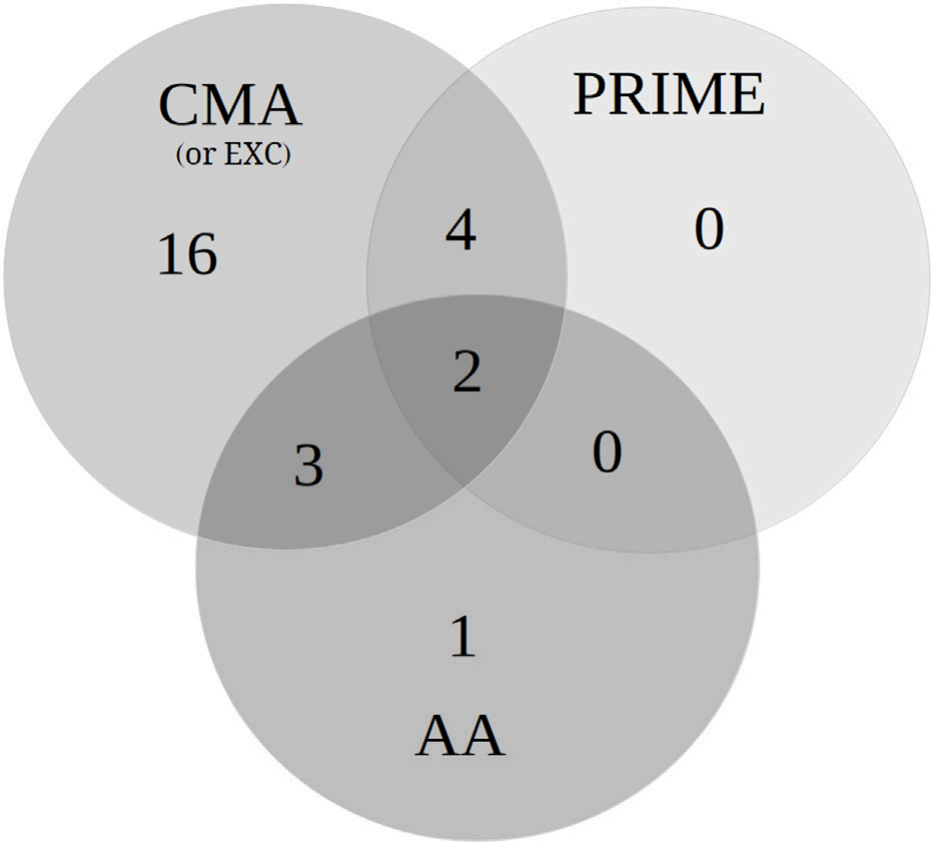
The Venn diagram of the number of MAAs granted with expedited program(s) (AA, CMA/EXC and/or PRIME).

Figure 5 and Table 1 present the different combinations (independent variables) of the MAAs granted with expedited program(s) (AA, CMA/EXC and/or PRIME) and/or OD status at the time the CHMP opinion was obtained. During the period analyzed, more than half of the MAAs (57%, n = 34) did not benefit from any expedited program at the time the CHMP opinion was issued. Conversely, 43% of the MAAs (n = 26) were under at least one expedited program (AA, CMA/EXC and/or PRIME) (Table 1). Among them, only 3% of the MAAs (n = 2) were granted all three designations (AA, CMA/EXC, and PRIME) (Figure 5). For those MAAs, the products were mAbs indicated for the treatment of multiple myeloma. Additionally, there were no MAA granted only with PRIME program. In relation to the OD status, 20% of the MAAs were granted with OD and at least one expedited program (Table 1), at the time the CHMP opinion was obtained. Furthermore, 37% of MAAs (n = 22) were not under OD status or any expedited program (i.e., PRIME, CMA/EXC, or AA).

**TABLE 1:** Analysis of Independent Variable(s) related to MAAs and expedited program(s) and/or OD status.

Independent variable (s)	% (Number of MAAS)
No expedited program (AA, CMA/EXC or PRIME)	57% (n = 34)
At least one expedited program (AA, CMA/EXC or PRIME)	43% (n = 26)
Under OD and at least one expedited program (AA, CMA/EXC or PRIME)	20% (n = 12)
Under OD but no expedited program (AA, CMA/EXC and PRIME)	20% (n = 12)
No OD and at least one expedited program (AA, CMA/EXC or PRIME)	23% (n = 14)
No OD and No expedited program (AA, CMA/EXC and PRIME)	37% (n = 22)

### 3.4 Analysis of LoQ response time

**FIGURE 6:**
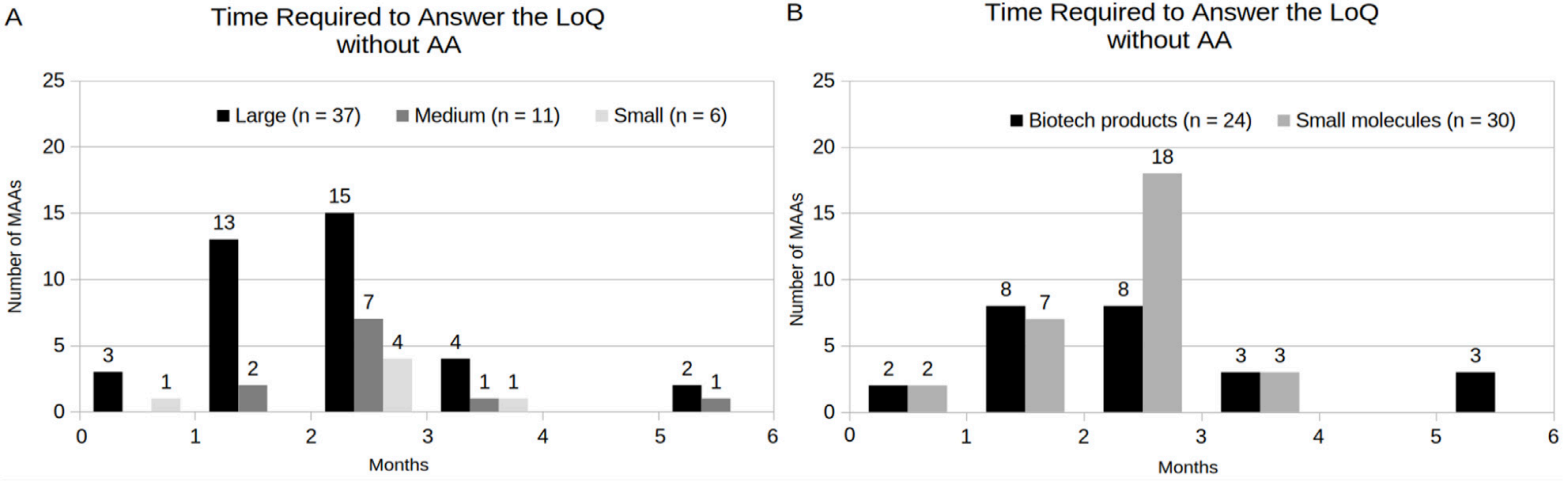
Time Required to Answer the LoQ of MAA under the standard procedure (without AA), by company size **(A,B)** by product category, respectively.

Figure 6 shows the response time of MAAs to the LoQ under a standard procedure, considering the following variables: company size and product category. 90% of the MAAs (n = 54) were not under the AA program. Among these, 83% MAAs (n = 45) submitted their responses to the LoQ before the last working day of the third month. The majority of these (69%, n = 31) were represented by MAAs with large-sized company holders and had small-molecule products involved (60%, n = 27). The remaining MAAs (17%, n = 9) required an extension, with response times ranging from four to 6 months. In relation to MAAs under the AA program, these applicants required less than 2 months to submit their responses to the LoQ. However, only 10% of the MAAs (n = 6) were under the AA program, which were represented by large-sized company holders and involved only biotechnology-related products (Supplementary Figure S1). Additionally, two MAAs were initially under the AA program during the LoQ stage but reverted to the standard procedure because the applicants requested an extension during the list of issues (LoI) phase (also known as the second clockstop) (data not shown). Consequently, they were not included among the MAAs with AA during LoQ.

**FIGURE 7:**
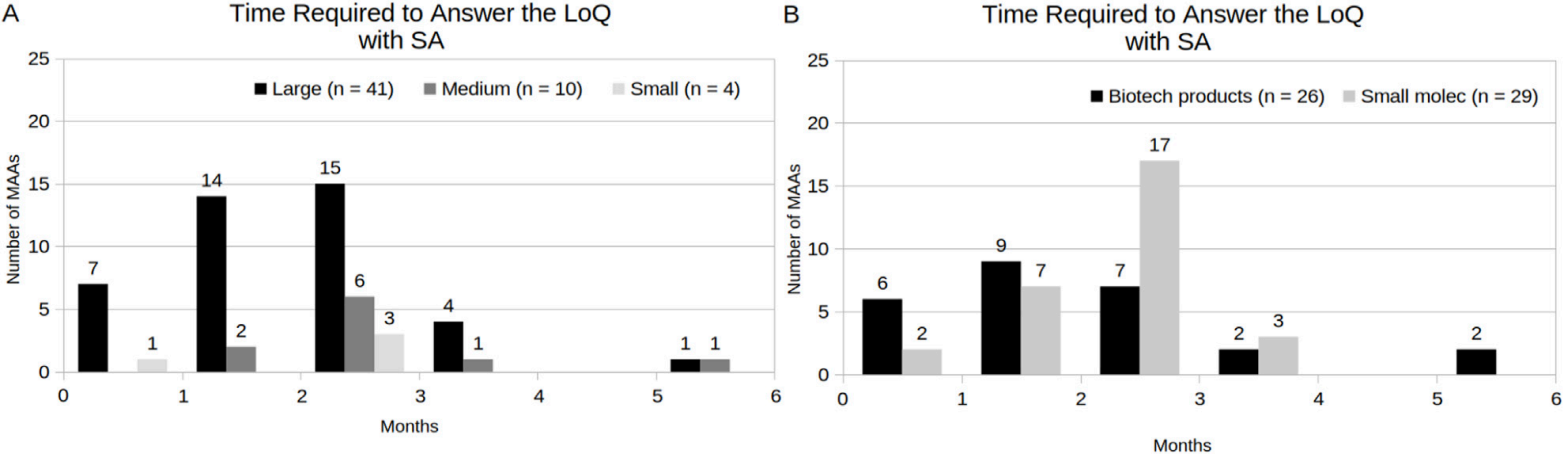
Time Required to Answer the LoQ of MAA with SA, by company size **(A)** and **(B)** by product category, respectively.

Figure 7 highlights the response time of MAAs that obtained SA to the LoQ, considering the following variables: company size and product category. Notably, the majority of the MAAs with a SA submitted their responses to the LoQ within 3 months (96%, n = 53), including medium- (16%, n = 9) and small-sized (7%, n = 4) company holders. Nevertheless, the predominant profile was composed by large-sized company holders (73%, n = 40) and involved small molecules (53%, n = 29) as products. Additionally, only 8% of the MAAs (n = 5) did not request SA. Among these, 40% (n = 2) were not able to submit their responses to the LoQ within 3 months (Supplementary Figure S2). In contrast, 87% of the MAAs that obtained SA (n = 48) have fulfilled this requisite.

### 3.5 Analysis of the procedural duration

**FIGURE 8:**
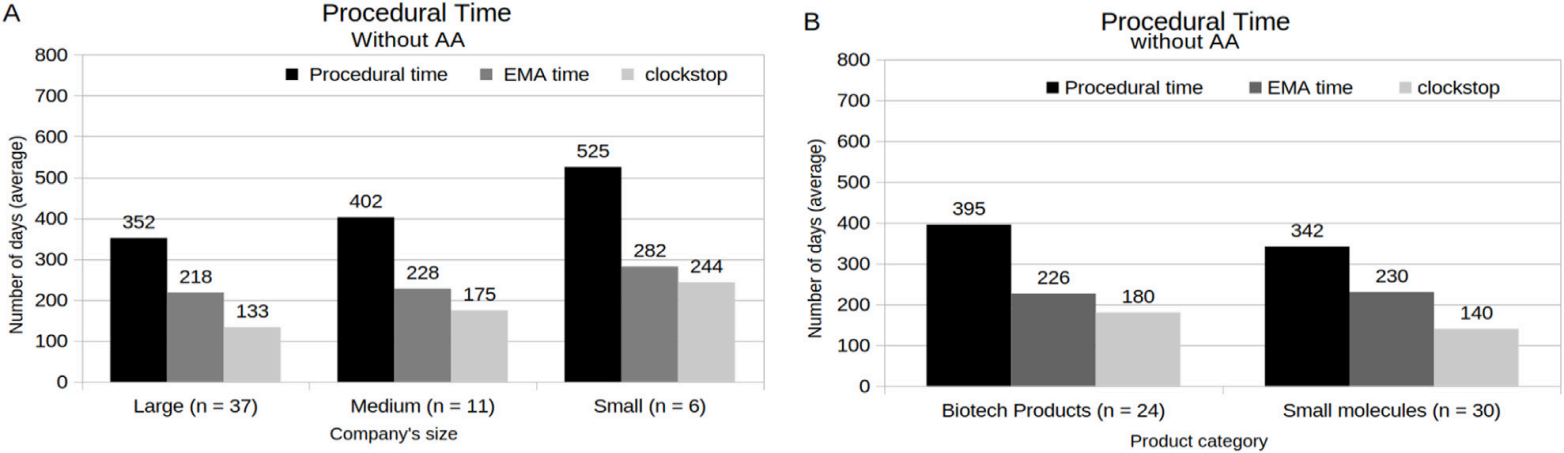
Analysis of the Procedural time under standard procedure time (without AA) by company size **(A)** and by Product category **(B)**.

**FIGURE 9:**
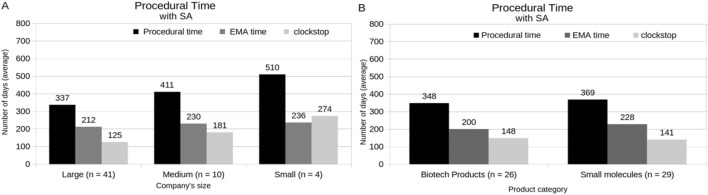
Analysis of the Procedural time of MAAs with SA, in relation to company size **(A)** and product category **(B)**.

Figure 8 demonstrates the MAA procedural time under a standard procedure considering holder’s and product’s features. Compared with MAAs with medium- (402 days) and small-sized (525 days) company holders, those MAAs with large-sized company holders had a short total procedural time, with an average of 352 days (Figure 8A). In relation to the product characteristics, the clockstop time appears as the main impact factor of the total procedural time (Figure 8B). Here, MAAs with small molecules involved showed a shorter average of the clockstop time, followed by a total procedural time of 342 days, in relation to the average (395 days) of MAAs with biotechnology-related products involved. In addition, MAAs under AA program, which were composed only by MAAs with large-sized company holders and biotechnology-related products involved, showed an average of 185 days (Supplementary Figure S3). When the procedural time of MAAs that obtained SA was analyzed, it was revealed that large-sized company holders showed an average total procedural time of 337 days, while medium- and small-sized company holders exhibited 411 and 510 days, respectively (Figure 9A). Among these MAAs, those with biotechnological products involved presented an average of total procedural time of 348 days, while those with small molecules involved showed an average of 369 days (Figure 9B). Additionally, except for MAAs from medium-sized company holders, all MAAs that obtained SA showed a shorter average procedural time when compared with those without SA (Supplementary Figure S4A). Similarly, procedural time was substantially reduced for MAAs with SA when analyzed by product category (biotechnology-related products without SA: from 500 days, and small molecules-based products without SA: from 399 days) (Supplementary Figure S4B).

## 4 Discussion

Over the past decade, drug discovery has advanced through the evolution of preclinical and clinical research, playing a pivotal role in translating biological insights into oncology therapeutic strategies (Al-Karmalawy et al., 2025). In view of this, the present study summarizes the key features of EU applications for new cancer therapy drugs over 5 years, focusing on regulatory insights, time-frame relevance, and evidence-based conclusions related to a critical health issue.

A previous study showed an average of 9.6 oncology applications per year in the EU during the period from 2010 to 2019 (Garsen et al., 2021). Here, it was possible to confirm a rise of 12.5% in the total number of MAAs in the last 5 years, with an average of 12 per year. The inclusion of regulatory programs and incentives aimed at accelerating procedural timelines can be considered a potentially attractive tool for pharmaceutical companies, which may, in turn, stimulate anti-cancer research. Furthermore, compared with the 2010–2019 period, the number of oncology MAAs receiving a positive CHMP opinion has increased markedly, from 89% to 98% in the present analysis. Conversely, the proportion of negative opinions decreased from 11% to 2%. This improvement may be associated with the increase of the quality and reliability of clinical development plans and scientific guidance by the use in SA, which rose from 81% to 92%. The primary purpose of SA is to assist medicine developers in generating sound and reliable evidence on a product’s efficacy and safety (Tavridou et al., 2020).

Drug discovery and development can take up to 10 years and might require large investments before reaching the patient (Berdigaliyev and Aljofan, 2020). Although small companies, together with academia, represent the main sources of oncology drug discovery, large-sized companies predominantly hold responsibility for the clinical development and process approval of these drugs (Kennedy et al., 2023). Similarly to Garsen et al. (2021), the data shown in this study revealed a relatively high proportion of 72% of large-sized companies as MAA holders. The correlation between large-sized company holders and positive application results implies that having ample resources and regulatory experience plays yet a crucial role in achieving marketing authorization approval (Regnstrom et al., 2010).

Among the therapeutic indications in oncology, the present study shows a rise of indications pertaining to blood cancers (from 32% to 54%), lung cancers (from 14% to 17%), and ovarian and peritoneal cancers (from 3% to 5%), respectively, when compared with the period of 2010–2019 reported in the literature (Garsen et al., 2021). On the other side, some cancer indications have decreased in relation to the previously mentioned period, such as skin cancers (from 15% to 5%), gastrointestinal and colorectal cancers (from 7% to 6%), prostate cancers (from 7% to 2%), and renal cancers (from 4% to 0%), respectively. Several types of cancers have low prevalence, and are therefore considered rare diseases. As a support for the development of treatments for rare diseases, OD drugs receive regulatory and financial incentives (Fermaglich and Miller, 2023). Although OD is supported by promising non-clinical and/or early clinical data, the efficacy and safety of these products remain to be established, and the later stages of development are often the most challenging (Mulder et al., 2021). The present study revealed that the most of the oncology MAAs (60%) applied to the marketing authorization with an OD status during the period analyzed. However, only 40% of the total of MAAs remained under OD at the moment of the CHMP opinion. This result is similar to what has been reported in the literature (Garsen et al., 2021). Concerning this, OD is typically removed when the marketing authorization holder requests it, often after the drug is approved and effective alternative therapies are available. In general, the OD withdrawal likely reflects a strategic regulatory decision due to market evolution together with insufficient clinical data to demonstrate a significant benefit over existing treatments, which were already available for the same therapeutic indications (Montanaro et al., 2021). According to Gomez-Ferreria et al. (2025), providing the EMA with additional resources and responsibilities could help retain existing developers of orphan medicines and attract new ones to the field. This, in turn, would likely boost clinical trial activity, facilitate market approvals, and improve the availability of orphan medicines throughout Member States.

Expedited programs, such as PRIME, CMA (or under EXC), and AA programs benefit MAAs by providing early regulatory guidance, accelerating development, enabling approval based on preliminary data, or shortening procedural timelines. In the present study, a rise was observed in the number of MAAs under PRIME (from 3% to 10%), CMA (from 24% to 42%), and AA (from 34% to 37%) programs when compared with literature data that analyzed the period from 2010 to 2019 (Garsen et al., 2021). Still relative to the previous period, a reduction (from 86% to 57%) was observed in the number of MAAs under AA that reverted to the standard procedural time. Furthermore, the proportion of MAAs with no expedited program (i.e., AA, CMA, and PRIME) exhibited a slight reduction (from 40% to 37%). All this together, likely reflecting improved dossier quality and more proactive engagement with the CHMP, which allowed applications to remain within the accelerated review timeline. In this regard, the experience factor, reflected by the predominance of large-sized companies as MAA holders, as well as the majority of MAA holders who have obtained SA, could be considered a decisive element related to the acquisition and maintenance of these programs by the end of the procedure.

Within the CP, the EMA aims to complete the evaluation of both original submissions in 210 active review days. This timeline excludes the periods when the review time is suspended to allow the applicant to address EMA inquiries, which often extends the total procedural time of the assessment process (EMA, 2025a). In the present study, the analysis of procedural time revealed that the size of the holding company, participation in the AA program, and SA might be considered factors influencing the reduction of total procedural time. However, only the AA program is directly related to a formal EMA time reduction, while SA only implies the general quality of the dossiers and applicant’s responses associated with CHMP questions, which, in turn, affects the clockstop time. Another component observed that affects the total procedural time is the time to answer the LoQ (as known as first clockstop). Similarly with Garsen et al. (2021), the present study shows that the majority of the MAAs under standard procedural time were able to address EMA inquiries within the required 3-month time-frame. Nonetheless, the proportion of MAAs with small molecules involved exhibited an increase during the period of 2010–2019 (from 69%), in relation to the period of 2020–2024 (to 90%), while the MAAs with biotechnological products involved showed a slight reduction (from 69% to 60%). The fact that large-sized companies formed the majority of the applications in the last 5 years may have contributed to this outcome. Likewise, the high number of SA meetings during drug development and the procedure assessment, might have contributed to higher dossiers quality in the last years.

In summary, the present study highlights the key features of oncology applications in the EU in the last 5 years. Moreover, despite the low number of MAAs under OD and expedited programs, especially in AA program, their importance in the quality of oncology products and in shortening patient access to these therapies is notable. In addition, SA has been shown as a helpful guidance tool during all the process, which might be contributed to reduce both the time of response to LoQ and the procedural time. However, this study has certain limitations, including the small number of MAAs with specific characteristics, what hindered suitable comparisons, and the reliance on publicly available data, which may not fully reflect unpublished regulatory interactions.

## 5 Conclusion

The number of oncology MAAs has increased over recent years, particularly for mAbs and blood cancer indications. The ongoing growth of oncology MAAs, particularly for biotechnology-related products, emphasizes a continued need for streamlined regulatory pathways that balance efficiency with rigorous assessment, ultimately facilitating faster patient access to innovative therapies, especially for rare diseases. The fact that large pharmaceutical companies represented the majority of applicants in the EU, together with SA, may be associated with shorter procedural times, highlighting the value of experience and guidance in expediting regulatory evaluations. These findings underscore the importance of early and structured regulatory engagement, particularly for medium- and small-sized companies, to optimize submission strategies and reduce delays.

## Data Availability

The datasets presented in this study can be found in online repositories. The names of the repository/repositories and accession number(s) can be found below: https://www.ema.europa.eu/en/medicines/download-medicine-data.

## Footnotes

1
https://www.ema.europa.eu/en/medicines/download-medicine-data

## Supplementary material

SUPPLEMENTARY FIGURE S1 Time Required to Answer the LoQ of MAA under AA, by company size **(A,B)** by product category, respectively.

SUPPLEMENTARY FIGURE S2 Time Required to Answer the LoQ of MAA without SA, by company size **(A,B)** by product category, respectively.

SUPPLEMENTARY FIGURE S3 Analysis of the Procedural time under AA, by company size **(A)** and by Product category **(B)**.

SUPPLEMENTARY FIGURE S4 Analysis of the Procedural time of MAAs without SA, in relation to company size **(A)** and product category **(B)**.
