# Supplementary figures and images for: Trends in oncology marketing applications in the European Union: a five‐year systematic review
Source: Front Pharmacol. 2026 Jan 20;16:1738701. doi: 10.3389/fphar.2025.1738701 (PMC12865204; doi:10.3389/fphar.2025.1738701)

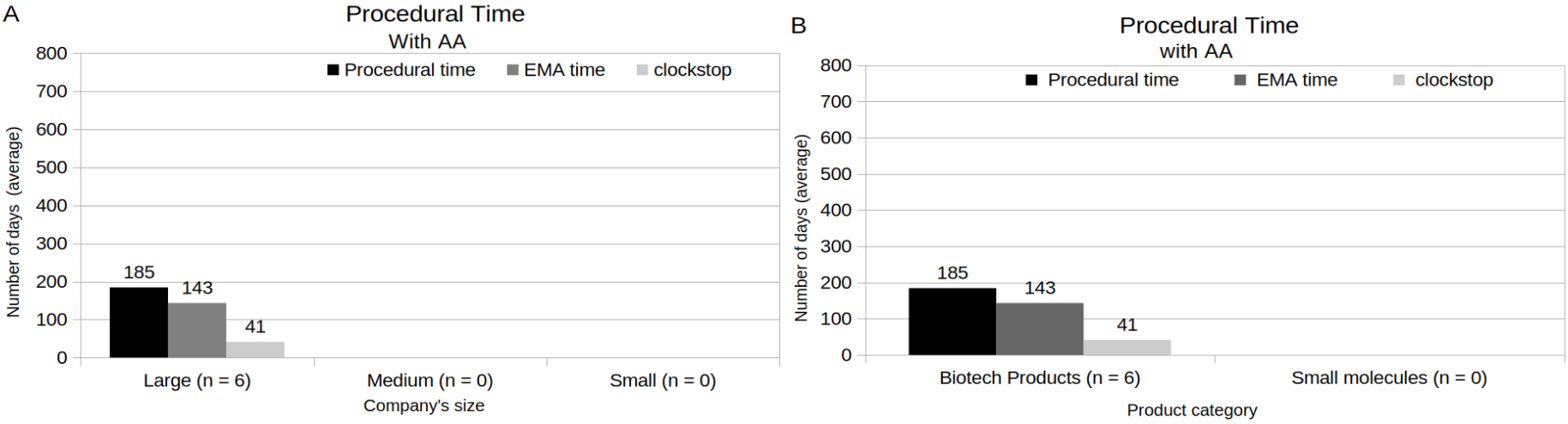

Supplement: Supplementary file 1 [file Image3.tif]

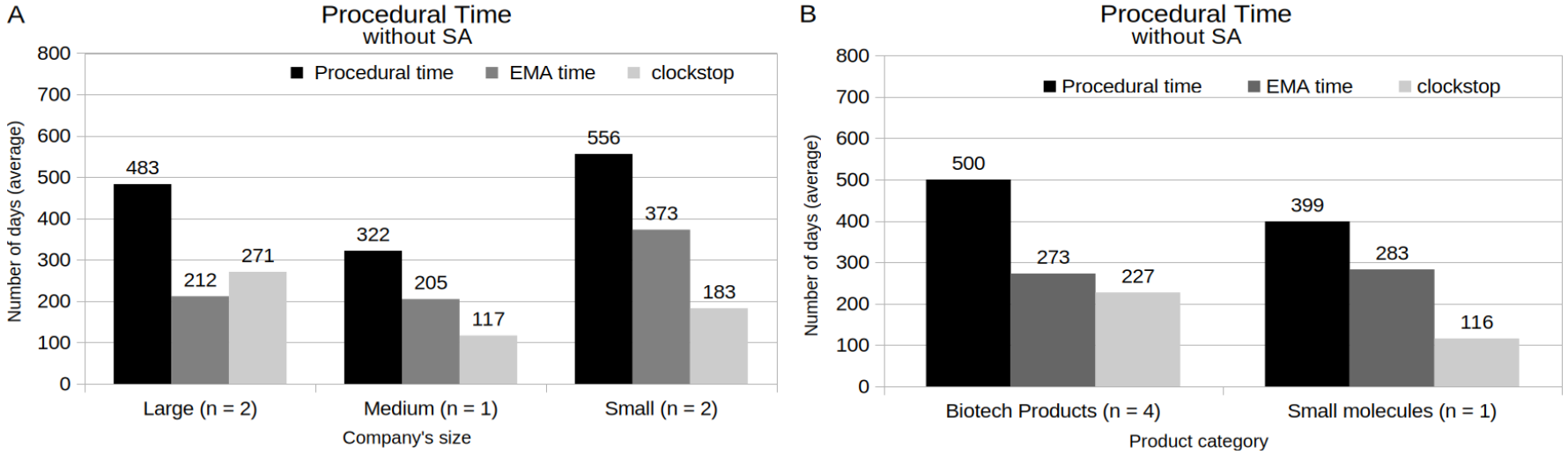

Supplement: Supplementary file 2 [file Image4.tif]

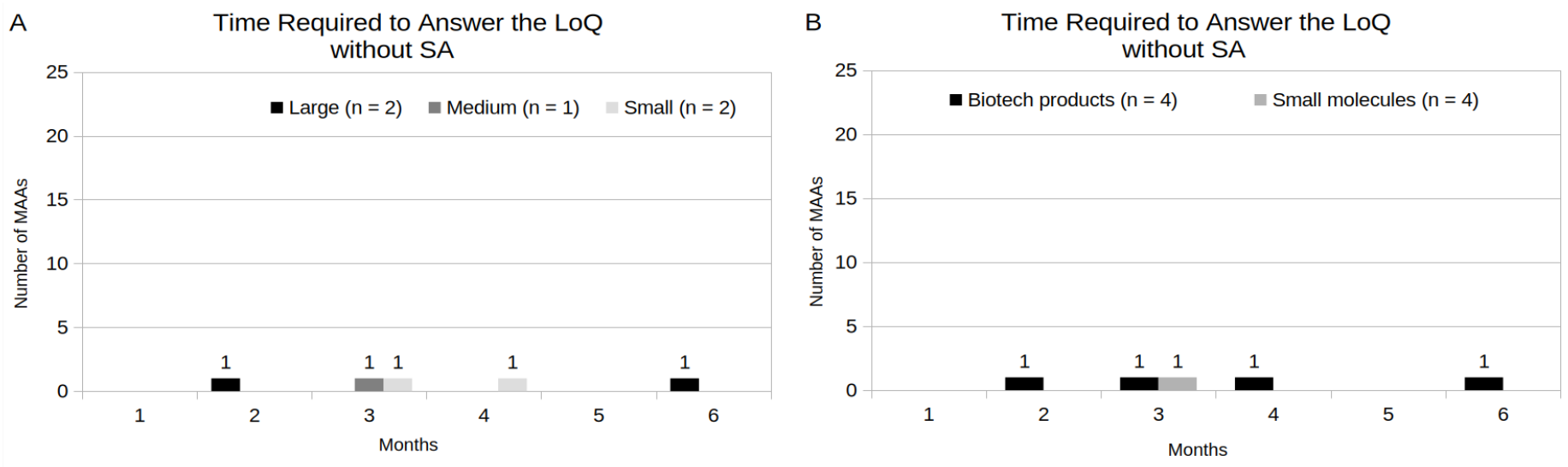

Supplement: Supplementary file 3 [file Image2.tif]

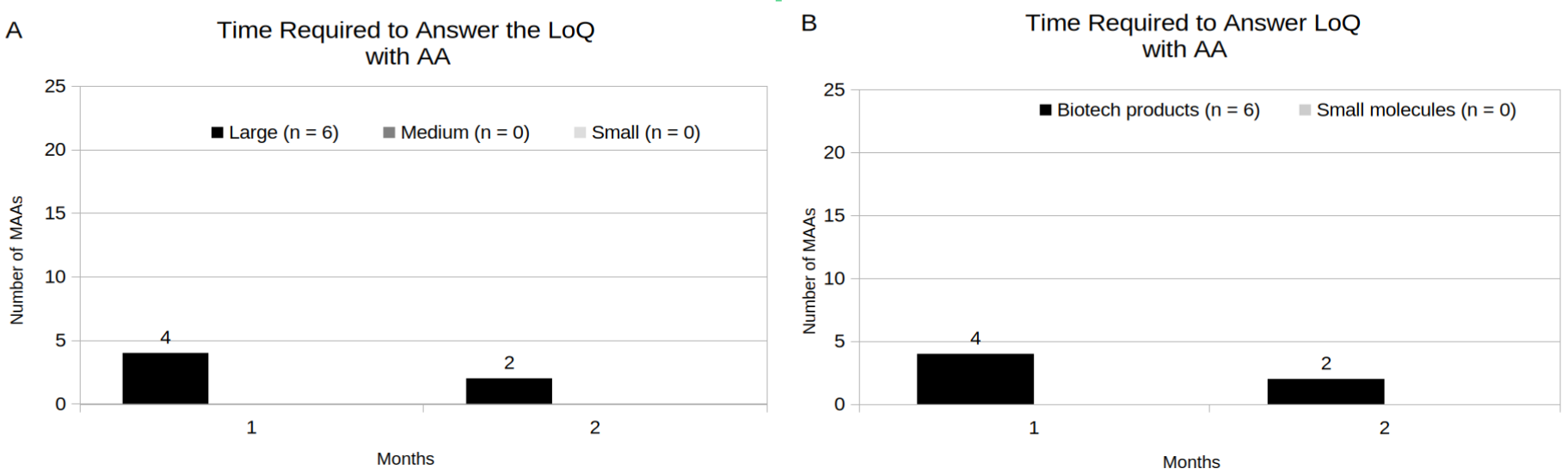

Supplement: Supplementary file 4 [file Image1.tif]
